# The Victim

**Published:** 1860-03

**Authors:** 


### THE VICTIM.

We have no time to study what subjects we shall treat of under the circumstances related on our first page, and as to remembering what we had written, that would be an impossibility. In five minutes the words of the pen are gone from the memory, utterly beyond reach.

But she was just eighteen, the only child of a retired merchant. Never was there a more indulgent father, never a more doating mother. That father had spent thirty long years bending over his desk. How sedulously had he made every entry! How late in the night of every day was it that he found himself running over his "blotter" to see if he had forgotten an item! How to the latest verge of conscience had he gone every Saturday night over the balance-sheets! How through wind and rain and storm and snow he had regularly "gone on" to purchase goods twice a year? How many heart-aches he had endured in that "age" of business, in the failure of customers to "pay up;" in their questioning the correctness of some of the entries; in listening to interminable excuses for want of promptness. How often did it happen, when after having done all that he could possibly do, to "meet his own notes," the announcement was made just before the clock struck "three," that he must "take up" a customer's paper, on the faith of which he had obtained a "discount," or go to protest? How many nights he had slept not a wink in the apprehension that he might not be able to meet the "calls" of the coming day? How many times he had come home at night-fall more dead than alive, hungry, tired, dispirited, and sad, soliloquizing, "What's the use of all this?" and yet, turning his eye on his patient, quiet, beautiful wife, and the more beautiful blossom which nestled by her side, would find a new inspiration in the thought: It's not for me, it's for these!

How many times such things occurred in the course of that thirty years of mercantile life, none can say; the number was doubtless large, very large. But the sun of prosperity shone in a cloudless sky. Money multiplied on itself; and at the age of fifty-eight, he found himself a rich man, retired from business, the owner of a splendid mansion, the husband of as good a wife, the father of as sweet a child as any reasonable man could wish to have. On the second day of June, eighteen hundred and fifty-eight, we were consulted as to the health of that daughter. She was at school in a distant city. The "examination" was coming on. She had maintained a high position in school. Hers was the glory of being at the "head of her class." Her ambition was to maintain that position to the end. On inquiry, it appeared that she was so much "interested in her studies" that she would not give any time to recreation. She would even take her food in her hands, hurry off to school, eating and studying on the way. The moment she returned from school, her face was buried in her books; and thus it had been for weeks, months, may be years. Great nature never allows an outrage against herself to be committed with impunity: neither youth nor beauty nor position nor gold ever bribed her; her laws are as immutable as adamant. The danger appeared imminent. It was counseled to abandon school. But as this was not assented to, we declined special advice. It was intimated that when the examination was over, (and it would only be a few weeks,) she could give full attention to herself. Not having seen her, we hoped that our fears were exaggerated. Still we felt as if every book had better be thrown in the fire; that not one single day should be allowed to be passed in a school-room, not an hour in study; that every moment in the beauteous out-doors was a treasure to her, and that the early morning and the later evening should find her in the saddle, scouring the hills of her own beautiful New-England. Only a few weeks! Why, it seemed to us, in its necessities, to be a million years' duration — in fact, an interminable time, irredeemable!

But she was anxious to graduate with honor. Parental kindness overreached itself. Moral firmness was wanting. And the school kept on. She graduated with great honor, and in the following June she died. The desolation of that household was immeasurable. "I see my error now," said the stricken father.

How many of our readers will take warning from this unvarnished narration of facts, and look with horror on those murderous stimulations of pride and ambition which are practised at almost all our schools? Practised always, to show off the teachers, without ever bringing one single benefit to the child. The price we pay for the education of our sons and daughters is, in ten thousand instances, the price of blood, paid for by the blasting of the hopes of a lifetime; the penalty, an age of desolation, a going down to the grave in an awful loneliness, for it is not merely to be alone, but the being attended with a remorse which death only can wipe out.

The victims to ill-advised applications at school and academy and college and seminary are numberless. Not, indeed, the applications themselves, but the injudicious habits and modes of life in connection with them.

We are all too much in a hurry to have our children graduate; to hasten their studies; to expedite their entrance on professional life, with the result of an utter failure; or if the professional goal is reached, let the experience of the myriads of sufferers from various forms of disease testify, which torture the body and harrass the mind for the remainder of life, making it a martyrdom instead of a glory a gladness and an enduring joy.

